# Gait in Benign Paroxysmal Positional Vertigo

**DOI:** 10.3389/fneur.2021.633393

**Published:** 2021-02-12

**Authors:** Yong-Hyun Lim, Kyunghun Kang, Ho-Won Lee, Ji-Soo Kim, Sung-Hee Kim

**Affiliations:** ^1^Department of Neurology, School of Medicine, Kyungpook National University, Kyungpook National University Chilgok Hospital, Daegu, South Korea; ^2^Center of Self-Organizing Software-Platform, Kyungpook National University, Daegu, South Korea; ^3^Department of Neurology, School of Medicine, Kyungpook National University, Daegu, South Korea; ^4^Brain Science and Engineering Institute, Kyungpook National University, Daegu, South Korea; ^5^Department of Neurology, Seoul National University College of Medicine, Seoul National University Bundang Hospital, Seoul, South Korea; ^6^Department of Neurology, School of Medicine, Ewha Womans University Mokdong Hospital, Seoul, South Korea

**Keywords:** vertigo, BPPV, gait, posture, vestibular

## Abstract

**Purpose:** Patients with benign paroxysmal positional vertigo (BPPV) experience gait unsteadiness not only during the attacks but also between the spells. This study aimed to measure gait changes in BPPV and determine whether these changes are associated with the involved canal or lesion side.

**Methods:** We recruited 33 patients with a diagnosis of unilateral BPPV. Patients with other vestibular or central nervous system disorders were excluded. Gait was assessed using the GAITRite™ system before and after canalith repositioning treatment (CRT).

**Results:** After CRT, improvements were observed in various gait parameters including velocity (*p* < 0.001), cadence (*p* < 0.001), functional ambulation profile (*p* = 0.011), and the coefficient of variation of stride time (*p* = 0.004). Exploration of the center of pressure (COP) distribution also revealed improved stabilization during locomotion after CRT. The spatiotemporal gait variables did not differ between the patients with horizontal- and posterior-canal BPPV, or between the ipsilesional and contralesional sides before CRT.

**Conclusions:** The gait parameters reflecting velocity and rhythmicity along with stability of COP distribution improved after the resolution of BPPV. Episodic overexcitation of semicircular canal may impair the vestibular information that is integrated with the other reference afferent systems and lead to impaired gait performance.

## Introduction

Despite problems with the posture and gait between vertigo episodes in BPPV having often been overlooked, there is increasing evidence that patients with BPPV indeed experience a deficit of postural control during a static stance when evaluated in posturographic studies (4, 5). Patients with BPPV exhibit significantly increased anterior–posterior sway in stance, which is decreased to different degrees after canalith repositioning treatment (CRT) (6). However, the dynamic gait performance in BPPV has rarely been explored. A study has evaluated the dynamic gait in BPPV based on the tandem walking speed (7), and a recent study demonstrated faster walking after CRP in 32 patients with posterior canal (PC) BPPV (8). The present study aimed to determine whether BPPV causes natural gait disturbance that may respond to CRT. We quantified the gait characteristics in patients with unilateral BPPV before and after CRT, and explored whether the gait patterns differ according to the involved canal type or lesion side.

## Materials and Methods

### Participants

BPPV was diagnosed based on the manifestation of brief positional vertigo episodes, presence of the typical positional nystagmus provoked by the Dix-Hallpike or supine rolling test, and absence of a neurological sign suggesting damage to the central nervous system. Nystagmus was observed and recorded with an infrared video system. Patients with other identifiable vestibular disorders including unilateral or bilateral vestibulopathy, Meniere's disease, or labyrinthitis based on abnormal findings in video head impulse tests, vestibular evoked myogenic potential tests, audiometry, and/or a caloric asymmetry of more than 25% were excluded. Patients with a history of migraine, stroke, recent head trauma, cardiovascular disease, cognitive impairment, or orthopedic problem were also excluded. The canalith repositioning maneuver was performed after the first gait assessment. Epley's maneuver was applied to patients with PC BPPV (9), while patients with horizontal canal (HC) BPPV received Lempert maneuver (barbeque rotation) for geotropic nystagmus (10) and Gufoni maneuver for apogeotropic nystagmus (11). The supine rolling test was repeated for the patients with apogeotropic HC BPPV 30 min later, and then the Lempert maneuver was executed for the patients who showed conversion of positional nystagmus into the geotropic variant.

### Evaluation of Gait

Each participant was instructed to look at the blank wall in front of them and to not move their head. The participant was instructed to walk barefoot and at a comfortable self-selected speed without a walking aid or cane. Patients were safeguarded by a researcher walking alongside them. The patients were told to walk at their preferred velocities without running and still safely. The platform along which the patients walked was 580 cm long and 89 cm wide. All data values were measured and calculated from raw data obtained using the GAITRite™ system (CIR Systems, Havertown, PA, USA) at a sampling rate of 120 Hz. The GAITRite system is a useful automated tool for measuring multiple spatiotemporal components of gait (12–14). The subjects walked twice in each condition, and the mean values were analyzed. The primary outcomes included temporal gait parameters (velocity, step time, swing time, stance time, cadence, single support time, and double support time), spatial parameters (stride length, step length, step width, and base support), functional ambulation profile (FAP), and the coefficients of variation (CVs) of stride time, stride length, and step width. The FAP score is an overall score based on the step length/extremity length ratio, step time, normalized velocity, and dynamic base support (15). The CV values reflecting gait variability were calculated using the standard deviations of stride time, stride length, and step width as determined from all of the steps recorded over the two passes. The CVs of the stride length and step width represent the gait fluctuation in the anterior-posterior and medial-lateral planes, respectively.

### Study Protocol

After an interval of about 1 week from CRT, each patient underwent a second comprehensive clinical evaluation to determine whether their BPPV had resolved. For patients who had residual vertigo or residual positional nystagmus, the canalith repositioning maneuver was repeated again, and a follow-up clinical evaluation was performed 1 week later. Only those patients who experienced the complete resolution of positional nystagmus and vertigo were asked to participate in the second gait assessment. The patients who had residual positional nystagmus or positional vertigo even after two cycles of CRT were excluded from the second gait evaluation. This study was performed in accordance with the Declaration of Helsinki and was approved by the local ethics committee. Written informed consent was obtained from each participant.

### Distribution of the Center of Pressure

**Figure 1:**
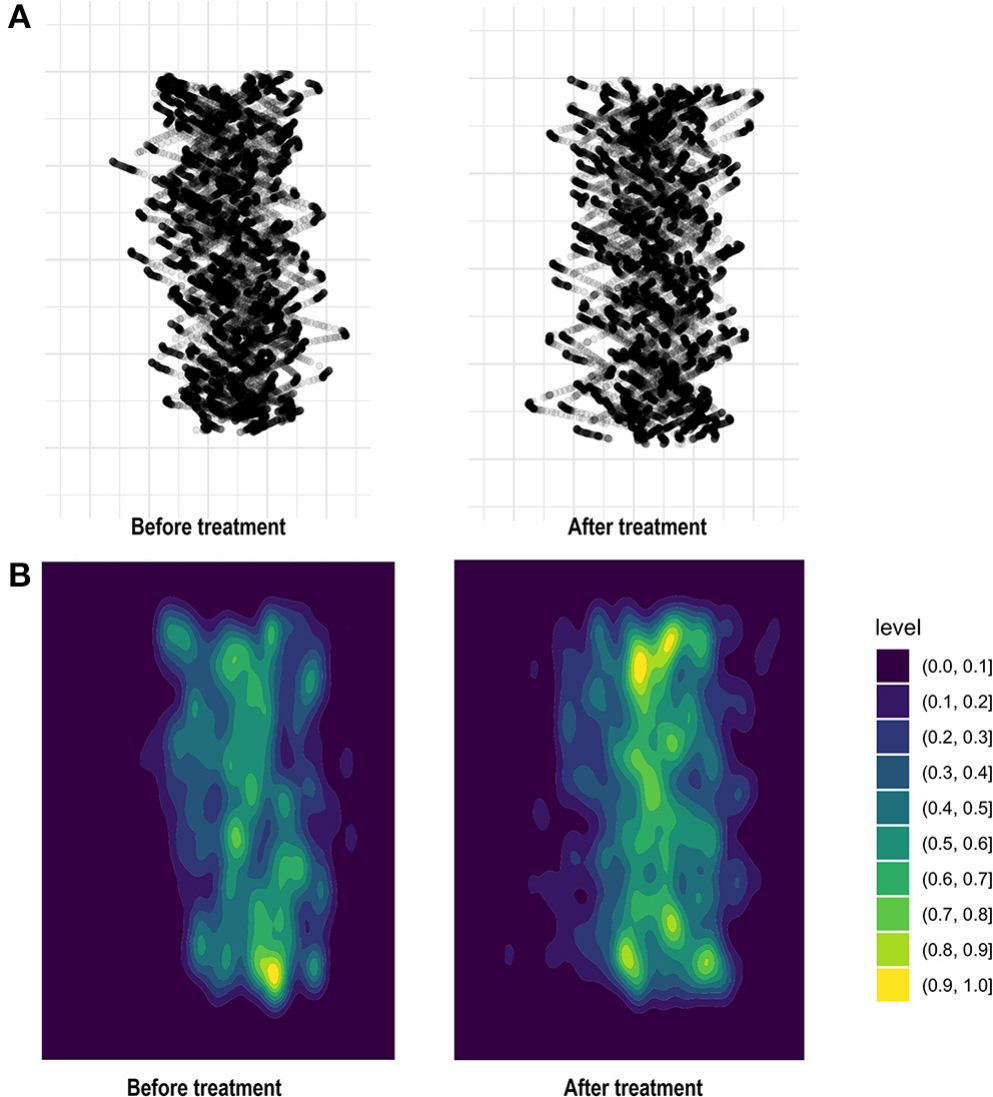
By tracking dots where the center of pressure (COP) falls throughout the gait platform, we aggregated a series of consecutive COP lines to produce a single black line **(A)**. Summated COP of 33 patients with BPPV visualized according to the summated density of COP lines, standardized to the range from 0.0 to 1.0 **(B)**.

Computer-aided gait analysis devices can measure pressure directly based on the changing distributions of force holding and moving body mass (16). By tracking the dots where the center of pressure (COP) was while walking on the gait platform, we obtained a series of consecutively measured COP lines from raw data in each patient. The series of COP lines was aggregated into a single composite line, as shown in Figure 1A. A COP line is thus a visual expression of the representative gait of a patient during which the foot is in contact with the ground. The summated COPs of every participant were visualized according to the summated density of COP lines, standardized to the range from 0.0 to 1.0.

### Statistical Analysis

Mean ± standard-deviation values of the dependent gait variables were calculated for the corresponding GAITRite raw data. Since the Shapiro-Wilk test demonstrated that the raw data satisfied the assumptions of population normality and homogeneity of variance, the data were analyzed parametrically. Comparisons between before and after CRT were performed using paired *t*-tests, while comparisons of gait between the HC and PC, and between ipsilesional and contralesional gait parameters before performing CRT, were performed using dependent *t*-tests. The criterion for statistical significance of individual gait parameters was *p* ≤ 0.05. Statistical analyses were performed using the software package SPSS (version 23, Chicago, IL, USA).

## Results

### Participants

This study recruited 33 patients with unilateral BPPV (age 60.20 ± 11.94 years, 23 females; Table 1), comprising 23 with PC BPPV (age 60.00 ± 12.75 years, 14 on the right side) and 10 with HC BPPV (age 58.50 ± 8.33 years, five on the right side). Six of the 10 patients with HC BPPV showed geotropic nystagmus, and the other four showed apogeotropic nystagmus. The intervals to the first gait assessments from symptom onset were 6.24 ± 9.15 and the intervals to the second gait from the first gait were 8.73 ± 5.94 days.

**Table 1:** Demographic characteristics of the 33 patients with benign paroxysmal positional vertigo (BPPV).

**Characteristic**	**Value**
Age (years)	60.20 ± 11.94
Sex, male / female	11/23
Involved canal, PC / HC	24/10
Lesion side, right / left	19/15
Interval to first gait evaluation from symptom onset (days)	6.24 ± 9.15
Interval to second gait evaluation from first gait evaluation (days)	8.73 ± 5.94

*PC, posterior canal; HC, horizontal canal*.

*Data are mean ± standard-deviation or n values*.

### Comparison of Gait Between Before and After Treatment

The following gait parameters changed significantly between before and after CRT: velocity (87.63 ± 19.85 to 99.77 ± 18.40 cm/sec, *p* < 0.001), cadence (101.81 ± 10.44 to 108.62 ± 7.26 steps/min, *p* < 0.001), FAP score (91.36 ± 10.04 to 95.18 ± 5.59, *p* = 0.011), and CV of stride time (5.05 ± 3.70% to 3.66 ± 1.83%, *p* = 0.004) (Figure 1). In contrast, there were no changes after CRT in base support (8.97 ± 2.16 to 8.81 ± 2.18 cm), CV of stride length (5.69 ± 3.72% to 4.54 ± 1.77%), or CV of step width (23.49 ± 13.70% to 22.41 ± 10.67%) (Table 2).

**Table 2:** Comparison of gait parameters in 33 patients with unilateral BPPV between before and after canalith repositioning treatment (CRT).

**Parameter**	**Before CRT**	**After CRT**	***p***
Velocity (cm/sec)	87.63 ± 19.85	99.77 ± 18.41	<0.001
Stride length (cm)	103.09 ± 17.27	110.39 ± 16.00	<0.001
Step length (cm)	51.24 ± 8.66	54.89 ± 8.05	<0.001
Single support time (sec)	0.43 ± 0.04	0.41 ± 0.03	0.002
Double support time (sec)	0.33 ± 0.09	0.28 ± 0.05	<0.001
Step time (sec)	0.60 ± 0.07	0.55 ± 0.04	<0.001
Swing time (sec)	0.43 ± 0.04	0.41 ± 0.03	0.002
Stance time (sec)	0.76 ± 0.10	0.69 ± 0.05	<0.001
Cadence (steps/min)	101.81 ± 10.44	108.62 ± 7.26	<0.001
CV of stride time (%)	5.05 ± 3.70	3.66 ± 1.83	0.004
Step width (cm)	51.25 ± 8.68	54.90 ± 8.12	<0.001
Base support (cm)	8.97 ± 2.16	8.81 ± 2.18	0.416
CV of step width (%)	23.48 ± 12.82	22.41 ± 10.67	0.796
CV of stride length (%)	5.69 ± 3.72	4.54 ± 1.77	0.127
FAP score	91.36 ± 10.04	95.18 ± 5.59	0.011

*CV, coefficient of variation; FAP, functional ambulation profile*.

### Comparison of Gait According to the Involved Canal Type or Lesion Side Before Treatment

The demographic characteristics of age, sex, and the interval between gait assessment and symptom onset did not differ between patients with HC and PC BPPV. The spatiotemporal gait variables measured before CRT did not differ significantly between the patients with HC and PC BPPV (Table 3).

**Table 3:** Comparison of gait parameters between patients with PC and HC BPPV before CRT.

**Parameter**	**PC, *n* = 23**	**HC, *n* = 10**	***p***
Velocity (cm/sec)	86.08 ± 22.03	91.18 ± 13.98	0.658
Stride length (cm)	100.91 ± 17.98	108.08 ± 15.59	0.475
Step length (cm)	50.21 ± 8.98	53.62 ± 7.77	0.307
Single support time (sec)	0.42 ± 0.04	0.43 ± 0.02	0.603
Double support time (sec)	0.34 ± 0.10	0.31 ± 0.04	0.832
Step time (sec)	0.59 ± 0.07	0.58 ± 0.02	0.686
Swing time (sec)	0.42 ± 0.04	0.43 ± 0.02	0.762
Stance time (sec)	0.76 ± 0.12	0.74 ± 0.03	0.556
Cadence (steps/min)	101.72 ± 12.34	102.02 ± 3.91	0.686
CV of stride time (%)	5.31 ± 4.25	4.43 ± 1.89	0.658
Step width (cm)	51.21 ± 8.98	53.62 ± 7.77	0.475
Base support (cm)	8.88 ± 2.39	9.16 ± 1.63	0.524
CV of step width (%)	25.54 ± 14.65	23.40 ± 11.86	0.237
CV of stride length (%)	5.44 ± 3.29	6.24 ± 4.19	0.923
FAP score	90.47 ± 10.59	93.40 ± 8.83	0.603

Before CRT, there were no differences in any spatiotemporal gait variables between the ipsilesional and contralesional sides (Table 4).

**Table 4:** Comparison between ipsilesional and contralesional gait parameters in 33 patients with unilateral BPPV before CRT.

**Parameter**	**Ipsilesional foot**	**Contralesional foot**	***p***
Stride length (cm)	103.22 ± 17.40	102.95 ± 17.39	0.611
Step length (cm)	51.43 ± 8.74	51.06 ± 8.75	0.398
Single support time (sec)	0.42 ± 0.03	0.42 ± 0.04	0.153
Double support time (sec)	0.33 ± 0.08	0.33 ± 0.08	0.298
Step time (sec)	0.59 ± 0.07	0.59 ± 0.07	0.158
Swing time (sec)	0.43 ± 0.04	0.42 ± 0.03	0.153
Stance time (sec)	0.75 ± 0.10	0.76 ± 0.10	0.336
Step width (cm)	51.43 ± 8.74	51.06 ± 8.75	0.406
Base support (cm)	8.88 ± 2.39	9.16 ± 1.63	0.249

### Distribution of the Center of Pressure Before and After Treatment

Figure 1B displays the COP distribution of 33 patients with BPPV according to the summated density of COP gait lines, standardized to the range from 0.0 to 1.0. The portions with lower density reflect that there were more COP gait lines that were easily shifted with lighter ground contacts, compared to the portions with higher density. When compared with COP distribution before treatment, there were increments in the portions with higher densities, which represented greater stability of force holding and moving body mass throughout the gait pathway after CRT.

## Discussion

This study found distinct differences in the gait characteristics of BPPV patients after CRT compared with before CRT. Our patients with unilateral BPPV showed a faster walking velocity, longer stride and step lengths, shorter swing and stance times, higher cadence, and decreased CV of stride time when BPPV was resolved. Analysis of the COP distribution also suggested improved stabilization during locomotion in our patients after CRT.

Gait function can be categorized into distinct domains, such as pace, rhythm, variability, and asymmetry (17). The gait pace is characterized by parameters that include gait speed, step length, and stride length, while rhythmicity during gait is characterized by swing time, stance time, and cadence (18). Our results showed that gait was impaired mainly in the domains of pace and rhythm in BPPV before treatment. In contrast, the spatial characteristics of gait such as base support, CV of stride length, and CV of step width did not change after the resolution of BPPV. Given that none of the spatiotemporal gait parameters differed significantly between PC and HC BPPV before CRT, the type of involved semicircular canal did not affect alterations in gait performance in BPPV before the treatment. The absence of significant differences in the ipsilesional or contralesional gait characteristics also rejected the assumption that our patients with unilateral BPPV show laterally deviated or asymmetric gait. Altered gait performance before resolution could not be attributed to the mechanical properties of BPPV such as the direction of the involved semicircular canal. Thus, abnormal vestibular signals caused by any movements of the otoconia debris in the involved semicircular canal during gait, if any, did not explain the impaired gait performance in BPPV.

Information from various sensory and motor systems is integrated to enable successful locomotion in humans. Vision is regarded as the most-salient afferent contributor to postural control and serves as a source of near-instantaneous feedback (19). Since vestibular afferents are sensitive to changes in the motion and position of the head, vestibular information is critical during more-dynamic states, such as when moving from standing to walking or when changing direction (20). Balance during locomotion also requires that the input of the vestibular system in the head be integrated with the somatosensory input from the feet (7). Impairment of the integration of sensory information—particularly of proprioception and vestibular graviception—may result in deficits of the internal model of postural verticality (21). While the altered gait in our patients may be partly attributed to the anxiety that is often induced by a vestibular disorder (22), consistent and detectable differences in various gait parameters achieved by the resolution of BPPV required a further explanation, such as disordered vestibular information, whether episodically occurred or not, possibly conflicting the visual and somatosensory spatial references required for stable postural control and effective locomotion. It is known that visual or vestibular perturbations alone can significantly impact walking (23). When one of these systems is perturbed, the unperturbed system (e.g., that providing more-reliable information) is given greater weighting (23). Sensory weighting is the ability of the central nervous system to weigh the degree of reliance on the primary modalities of sensory feedback for postural control (24). The relative weight assigned to each sensory system varies with the complexity of the postural task, environmental conditions, and fidelity of the input (24). Therefore, impaired fidelity of vestibular information in BPPV caused by paroxysmal overexcitation of the semicircular canal according to changes in head position may disrupt the normal integration of the vestibular input with the visual and somatosensory afferents into the body efferent system during gait.

Of note, some patients who develop persistent postural-perceptual dizziness (PPPD) may show a slow or hesitant gait pattern after resolution of BPPV. Most cases of PPPD are triggered by an acute vestibular disorder such as BPPV (25). Although the exact pathophysiology of PPPD remains to be elucidated, our explanation of sensory reweighting for gait alteration in BPPV seems to be in accordance with the pathophysiology of PPPD. Normally, alternative systems of movement control that are independent of vestibular system are activated to compensate for unreliable vestibular information until a vestibular disorder is recovered (25). In PPPD, however, the normal compensation is replaced by maladaptation that is especially provoked by an upright posture, visual stimuli, and motion. The patients who develop PPPD adhere to high-risk postural-gait control strategies with a higher reliance on visual input even after BPPV is resolved (26).

The current study did not intend to overstate the impairment of gait in BPPV. Despite slowness and impaired rhythmicity of walking, each of the included participants with unilateral BPPV could repeat the tests of gait performance without assistance before CRT. If a patient who has positional nystagmus suggestive of BPPV exhibits gait disturbance that results in significant dependency or a remarkably deviated trajectory, or the gait instability does not improve after the resolution of BPPV, then they should be examined carefully for the need to perform further imaging studies. Meanwhile, since our study has limitation of the relatively small participants, future studies with larger numbers are needed to identify the gait pattern according to the involved semicircular canal type.

Our study quantified changes in the gait of patients with BPPV and found that the gait parameters reflecting velocity and rhythmicity along with stability of the COP distribution improved after the resolution of BPPV. Even without direct injury resulting in static vestibular imbalance or defects in the vestibulo-ocular reflex, episodic overexcitation of a semicircular canal may cause infidelity of the vestibular information that is integrated with the other reference afferent systems and lead to impaired gait performance.

## Data Availability Statement

The raw data supporting the conclusions of this article will be made available by the authors, without undue reservation.

## Ethics Statement

The studies involving human participants were reviewed and approved by Institutional Review Board of Kyungpook National University Chilgok Hospital. The patients/participants provided their written informed consent to participate in this study.

## Author Contributions

Y-HL conducted the study and prepared the manuscript. KK and H-WL contributed to conduction of study and collection of data. J-SK revised the manuscript. S-HK created the concept, interpreted data, wrote the manuscript, and supervised the study. All authors contributed to the article and approved the submitted version.

## Conflict of Interest

The authors declare that the research was conducted in the absence of any commercial or financial relationships that could be construed as a potential conflict of interest.
